# Promoting mobility after hip fracture (ProMo): study protocol and selected baseline results of a year-long randomized controlled trial among community-dwelling older people

**DOI:** 10.1186/1471-2474-12-277

**Published:** 2011-12-07

**Authors:** Sarianna Sipilä, Anu Salpakoski, Johanna Edgren, Ari Heinonen, Markku A Kauppinen, Marja Arkela-Kautiainen, Sanna E Sihvonen, Maija Pesola, Taina Rantanen, Mauri Kallinen

**Affiliations:** 1 Gerontology Research Centre, University of Jyväskylä, Jyväskylä, Finland; 2 Department of Health Sciences, University of Jyväskylä, Jyväskylä, Finland; 3 Department of Physiotherapy, Central Finland Central Hospital, Jyväskylä, Finland; 4 JAMK University of Applied Sciences, Jyväskylä, Finland; 5 Department of Orthopedics and Traumatology, Central Finland Central Hospital, Jyväskylä, Finland; 6 Department of Physical Medicine and Rehabilitation, Central Finland Central Hospital, Jyväskylä, Finland

## Abstract

**Background:**

To cope at their homes, community-dwelling older people surviving a hip fracture need a sufficient amount of functional ability and mobility. There is a lack of evidence on the best practices supporting recovery after hip fracture. The purpose of this article is to describe the design, intervention and demographic baseline results of a study investigating the effects of a rehabilitation program aiming to restore mobility and functional capacity among community-dwelling participants after hip fracture.

**Methods/Design:**

Population-based sample of over 60-year-old community-dwelling men and women operated for hip fracture (n = 81, mean age 79 years, 78% were women) participated in this study and were randomly allocated into control (Standard Care) and ProMo intervention groups on average 10 weeks post fracture and 6 weeks after discharged to home. Standard Care included written home exercise program with 5-7 exercises for lower limbs. Of all participants, 12 got a referral to physiotherapy. After discharged to home, only 50% adhered to Standard Care. None of the participants were followed-up for Standard Care or mobility recovery. ProMo-intervention included Standard Care and a year-long program including evaluation/modification of environmental hazards, guidance for safe walking, pain management, progressive home exercise program and physical activity counseling. Measurements included a comprehensive battery of laboratory tests and self-report on mobility limitation, disability, physical functional capacity and health as well as assessments for the key prerequisites for mobility, disability and functional capacity. All assessments were performed blinded at the research laboratory. No significant differences were observed between intervention and control groups in any of the demographic variables.

**Discussion:**

Ten weeks post hip fracture only half of the participants were compliant to Standard Care. No follow-up for Standard Care or mobility recovery occurred. There is a need for rehabilitation and follow-up for mobility recovery after hip fracture. However, the effectiveness of the ProMo program can only be assessed at the end of the study.

**Trial registration:**

Current Controlled Trials ISRCTN53680197

## Background

Fall-related injuries leading to hospitalization and activity restriction result in adverse health outcomes, mobility limitation and disability which may last years or become permanent [1-3]. For older people, hip fractures are among the most severe consequences of falls [4,5]. Hip fractures cause considerable health care costs during the first post fracture year [6-8]. The cost burden will double or even triple with the subsequent fall and fracture particularly if a home-dwelling person is admitted to permanent institutional care because of the fracture [8,9].

Community-dwelling older persons who survive a fracture need special attention. To cope at their homes safely sufficient mobility and functional ability is needed. Only 40% of hip fracture survivors recover to their pre-fracture ambulatory level and only 20% recover to the pre-fracture level in advanced mobility tasks [3,10]. Safe mobility and participation are challenged by persistent pain [11,12], fear of falling and balance impairments [13,14], lower limb muscle weakness [11], reduced bone mass and impaired bone geometry [15]. Consequently, older community-dwelling people recovering from a hip fracture are at an increased risk for a new fracture, persistent mobility limitation and disability as well as loss of independence in the near future.

Currently there is insufficient evidence on the best practices supporting recovery after hip fracture [16]. The current research knowledge is mostly based on efficacy driven research in which the effects of highly specified short-term interventions without follow-up have been investigated among a homogenous group of hip fracture participants. These studies have been performed under optimal conditions with specifically designed and arranged training protocols and facilities. Previous efficacy studies have shown that rehabilitation programs including intensive and supervised training sessions with resistance and balance training improve mobility [17-20], physical functioning [18-21] and level of physical activity [22] among older community living persons who have suffered a hip fracture. However, the effects on mobility disability remain unclear.

A rehabilitation program that produces significant effects in an efficacy study may not have same effects under real-world conditions [23]. Moreover, persons who are likely to benefit the most from a program including physical activity are usually excluded from these studies. Travelling to organized and supervised sessions on a weekly basis in a gym with the necessary set-up may be too demanding for many fracture patients [19,24]. Therefore, rehabilitation programs aiming to restore mobility after hip fracture need to be implemented and studied in the real-world conditions or close to that. Moreover, participants should not be excluded unless there is an empirical or ethical reason to do so (e.g. possibility for negative side effect of training or main outcomes are impossible to measure) [25]. Home-based individually tailored rehabilitation programs including weight-bearing exercises [26,27], exercises with progressive resistance [28] and a systematic follow-up and support [22] may form the most promising approach to increase the effectiveness of the rehabilitation to prevent mobility disability after hip fracture.

The Promoting Mobility after Hip Fracture (ProMo) study investigates the effects of a year-long individually tailored and home-based rehabilitation program compared to the Standard Care on mobility recovery, physical functional capacity and disability among over 60-year-old community-dwelling men and women who suffered a proximal femoral fracture. The purpose of this article is to describe the recruitment process, design and intervention as well as to present demographic baseline results of this randomized controlled trial.

## Methods/Design

### Context

All Finnish residents have health insurance. In Finland, municipalities are responsible for organizing specialized and primary health care for all people. For example, in Central Finland specialized care is provided by the Central Finland Central Hospital for 23 municipalities with a total population of 273 700. Each municipality organizes primary health care including inpatient rehabilitation, ward care and outpatient clinic for their residents at the local health care centers. After a proximal femoral fracture, patients living in Central Finland are operated at the Central Finland Central Hospital and transferred to the local health care centre of their municipality for inpatient care and rehabilitation typically within the first post-operative days. The inpatient rehabilitation period ranges from one week to few months depending on the health status and care needs.

### Design

This study is a randomized controlled trial (RCT, ISRCTN53680197). Random allocation to the intervention (ProMo) and control (Standard Care) groups were performed after baseline measurements by the statistician, who was blinded to the study participants and their characteristics. The study group assignments were enclosed in sealed envelopes. Men and women and those operated with internal fixation or arthroplasty were randomized by blocks.

**Figure 1:**
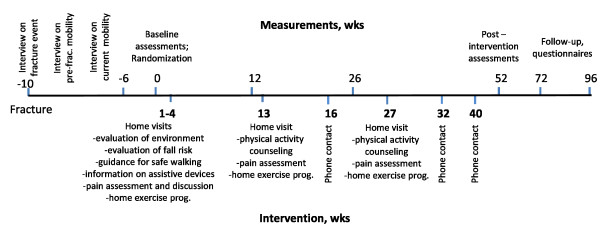
**Study design**.

All participants were measured at the laboratory four times; at baseline, three, six and 12 months. After that all participants were followed-up for an additional year to collect data on form of dwelling, mobility limitation, physical functional capacity, mood and quality of life with a structured questionnaire. Study design is described in detail in Figure 1.

Pretrial power calculations were based on previously published longitudinal data on mobility recovery after hip fracture. In the study by Visser et al, 45.3% of the community-dwelling participants were independent in more demanding mobility tasks (chair rising, walking one block and negotiating stairs) before the fracture [10]. Twelve months after hip fracture less than half of them (20.7% of the total sample) had regained their pre-fracture level of mobility. The purpose of our study was to restore the pre-fracture level of mobility by the ProMo rehabilitation program. To detect the expected difference (based on percentages 45.3 and 20.7 from the study by Visser et al) between the study groups in mobility recovery at α = 0.05 and β = 0.20, a minimum of 44 subjects was needed in each study group. Sample size was calculated using an online sample size calculator available from (DSS researcher's toolkit, http://www.dssresearch.com/KnowledgeCenter/toolkitcalculators/samplesizecalculators.aspx)

### Participants and recruitment

**Figure 2:**
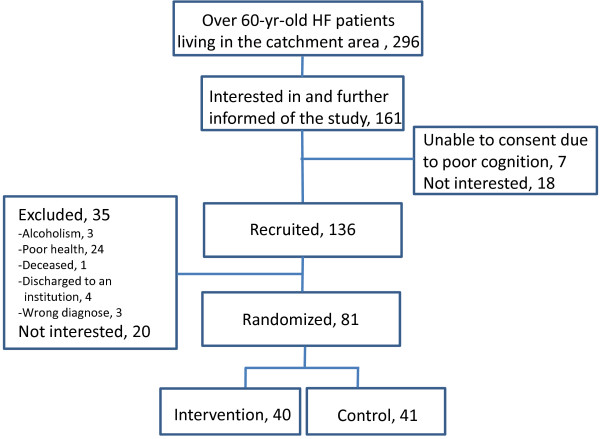
**Participant recruitment flowchart**.

Staff of the physiotherapy department of the Central Finland Central Hospital reviewed the medical records of all consecutive, over 60-year-old, ambulatory and community-dwelling men and women operated for femoral neck or pertrochanteric fracture (ICD code S72.0 or S72.1, http://www.cdc.gov/nchs/icd.htm) between 1.3.2008 and 31.12.2010 and living in the city of Jyväskylä or in nine neighboring municipalities. All patients fulfilling the inclusion criteria got an information letter on the study (n = 296). Of them, 161 patients expressed their initial interest in the study and were further visited by the ProMo representative during the inpatient period at the health care centre. Finally, 136 persons were recruited to the study. Patients living in an institution or confined to bed at the time of the fracture, suffering from severe memory problems (Mini Mental State Examination, MMSE < 18), alcoholism, severe cardiovascular, pulmonary or progressive (i.e neoplasm, ALS) disease, para-or tetraplegic or severe depression (Beck Depression Inventory BDI-II > 29) were excluded from the study. In total, 18 men and 63 women participated in the study. The flow chart of the study is shown in Figure 2.

### Ethical issues

This project was approved by the Ethics Committee of the Central Finland Health Care District on December 18, 2007 (11/2007). Written information on the study was given to all participants. Participants signed an informed consent prior to participation. Proxy consent was not permitted. Those who were interested in the study had an opportunity to discuss with the researcher before signing the informed consent and giving a permission to review their medical records.

### Measurements

Measurements and analysis will be performed blinded to the study group. Baseline measurements were organized as soon as possible after discharged to home; on average 70 (SD28) days after the hip fracture, 65 (21) days after the hip fracture operation and 42 (23, range 4-153) days after discharge to home. Measurements included a comprehensive battery of laboratory tests and self-report on mobility limitation, disability, physical functional capacity and health, as well as assessments for the key prerequisites of mobility, disability and functional capacity. All assessments were performed at the research laboratory.

#### Review of medical data and health status

Each participant was interviewed within 24 hours of the hip fracture with structured questions on the characteristics of the accident [29]. At baseline, during a medical examination performed by a nurse practitioner and a physician, the presence of chronic conditions, use of prescription medication, fracture status and date, type and date of surgery and lowest post-operation hemoglobin level during hospitalization were confirmed according to a pre-structured questionnaire, current prescriptions and medical records obtained from the local hospital and health care centers. To ascertain safe participation in the measurements and intervention, the physician evaluated contraindications according to ACSM guidelines [30] and acute conditions such as infections (e.g. acute respiratory or urinary tract infection) by blood count, C-reactive protein (hCRP) and hemoglobin (Hb) analysis. Cognitive status was assessed by Mini Mental State Examination (MMSE) [31], and depressive mood by Beck Depression Inventory (BDI-II) [32] at baseline. Self-rated health was determined by the question ''How would you describe your health?'' using a 4-point scale (very good, good, poor and very poor). Offending musculoskeletal pain in the low back, hip, knee, ankle and foot was assessed by a questionnaire. The question for the musculoskeletal pain was "Have you suffered from pain in the low back, hip, knee, ankle and foot region daily during the preceding month? Has the pain compromised your mobility?" Three alternative response options were: 1) no 2) yes, but the pain does not limit mobility 3) yes, the pain limits mobility.

#### Demographics, physical characteristics and living habits

Demographics included age, sex, living conditions, income and education. Body height and weight were measured using standard procedures and body mass index was calculated (body weight, kg/body height, m^2^*100). Body composition was assessed with Bioimpedance devise with eight polar electrodes (BC-418, TANITA, Tokyo, Japan). Maximal hand grip strength was measured from the dominant hand with a dynamometer (Metitur Ltd, Palokka, Finland) and bone density and geometry with a peripheral computed tomography [15]. Current level of physical activity was assessed by a standardized question with slight modifications [33]. The question included seven alternative responses: 1) mainly resting 2) most activities performed sitting down 3) light physical activity twice a week at the most 4) moderate physical activity about 3 h a week 5) moderate physical activity at least 4 h a week or heavy physical activity ≤ 4 h a week 6) physical exercise or heavy leisure time activity several times a week and 7) competitive sports several times a week. No one reported participation in competitive sports. Responses were categorized as sedentary (1 to 3) and active (4 to 6). Smoking status was assessed with a questionnaire (never, former, current smoker).

#### Main outcomes

The short term primary outcome (at 3 and 6 mo) is Short Physical Performance Battery (SPPB) including 2.44 m habitual walking speed, five chair rises timed and standing balance tests [34]. One year primary outcome will be mobility limitation assessed by interviewing the subjects for the ability to getting in and out of bed, rising from a chair, walking across a room, walking one block, and climbing stairs [10]. In addition, self-reports of perceived difficulty in walking outdoors, walking 500 m, walking 2 km and climbing one flight of stairs will be assessed by a questionnaire. Secondary outcomes include physical disability (validated questionnaire) [35], health related quality of life (RAND-36), walking speed over 10 meters [36], isometric knee extension strength for both legs [36] (Metitur Ltd, Palokka, Finland), leg extension power with the Power Rig for both legs [11], functional balance (Berg Balance Scale) [37] and fear of falling (Activities-specific Balance Confidence scale) [38]. Information concerning use of formal and informal care and form of dwelling will be collected by a questionnaire.

### Quality assurance

Our research centre has a long tradition and established methods on mobility and functional capacity assessments among older populations. A standard operation procedure was written before launching the study and then followed up carefully throughout the study. A system with periodical meetings and checks was set up for monitoring the quality of data collection. The personnel performing the measurements were carefully educated by a senior researcher. The same staff engaged in the data collection throughout the study except for the nurse practitioner who was replaced twice during the study. During the laboratory visits, all questionnaires were reviewed by the study coordinator. In case of missing information participants were asked to complete the questionnaire. If the participant was unable to come to the laboratory measurements at some point, self-reports were collected and Short Physical Performance Battery was performed at participants home.

### Control condition; Standard care

At baseline, information on standard care after the hip fracture was collected by interviewing all participants with structured questions on advice and recommendations concerning rehabilitation they had received at discharge from hospital and/or health care centre. Seventy percent of all participants obtained a written home exercise program with no difference between the intervention and the control groups (68% vs. 71%, p = 0.813). From those who received a home exercise program, 70% exercised every day, 21% on weekly basis and 9% few times a month or not at all. Typically the program included five to seven exercises including ankle flexion and extension, knee flexion and extension, and hip abduction and extension in supine, sitting and/or standing positions with no additional resistance. None of the participants were followed up for the home exercises and the program was not updated. Of all participants 12 received a referral to physiotherapy (5 in the intervention and 7 in the control group) while the rest did not get any further instructions regarding rehabilitation.

### ProMo -Intervention

Intervention includes standard care and ProMo-program which aims to restore mobility after hip fracture. The intervention starts after baseline measurements (Figure 1). ProMo is an individually tailored year-long physical activity and rehabilitation intervention taking place at participants' homes. It includes five to seven home visits by an experienced physiotherapist; the first three visits will be performed within one month followed by a visit three and six months after baseline measurements. If necessary, an additional visit two months after baseline will be performed. The scientific basis for the ProMo arises from a previous systematic review on fall and fracture prevention [39] and interventions that successfully prevented functional decline [26,40] among community-dwelling high risk groups of older people. ProMo comprises two partly overlapping phases. Phase I prepares the basis for the physical rehabilitation and physical activity. The content of Phase I is as follows: 1) Evaluation and modification of environmental hazards known to increase falls risk [41] 2) Guidance for safe walking including readjustment of walking aids and information on shoes and anti-slip shoe devices for icy conditions. In addition, written information on assistive devices and a brochure on hip protectors will be provided. All above mentioned information and brochures have been published before and are available for laypersons as well as for health care professionals 3) Pain assessment and discussions on pain relief strategies that the participants have perceived effective. Regular pain assessment and information on pain management are independently associated with better pain relief in hospitalized patients [42]. Pain assessments will be repeated three and six months after baseline measurements.

Phase II includes a progressive home exercise program and physical activity counseling. The exercise program comprises strengthening exercises for the lower limbs, balance training in standing position, walking exercises and stretching. The program will be delivered during the second home visit and it will be updated to a more challenging one during each following home visit. Accordingly, during the ProMo -intervention five written, progressive and individually tailored home exercise programs of approximately 30 minutes duration and designed by the PhysioTools software (PhysioTools, Tampere, Finland) will be delivered. Strengthening and stretching exercises will be performed three times a week and balance and walking exercises on two to three other days in the week. During the intervention, the resistance for the strengthening exercises will be individually increased with resistance bands with three different strengths.

An individual motivational face-to-face physical activity counseling session [43,44] will be scheduled approximately three months after the baseline measurements. The average duration of this session is 30 minutes. The topics covered during the counseling session include the level of physical activity and participation in physical exercise before the fracture, the persons' interest in returning to previous activities, beginning physical activity or exercise, the willingness to be active in everyday chores, to exercise on one's own or to participate in supervised exercise classes. The problem-solving method will be used to address perceived obstacles to physical activity and to access exercise facilities offered by the municipality. Pre-existing written information on the supervised physical activity classes and exercise facilities offered by the municipality will be given. Based on this information, the participant and the physiotherapist together design a personal physical activity plan, which will be signed during the session. After the first face-to-face counseling session, the physiotherapist will support compliance to the program and the behavior change through three phone contacts and one face-to-face session with 1-2 months interval. All participants in the intervention group will keep a physical activity diary on home exercises and physical activities.

### Data analysis

Means, standard deviations and frequencies for the demographic variables were calculated. Normality of the distributions was tested with Shapiro-Wilkinson test. The significance of differences between the intervention and control group was tested by cross-tabulation and chi-square tests in the case of discrete variables, by Student's t-test for independent samples for normally distributed data and by Mann-Whitney U-test for non-normally distributed continuous data. Association between variables was analyzed using Pearson correlation coefficient. Determinants for mobility limitation and physical disability will be assessed by linear and logistic regression analysis and the theoretical pathway to mobility limitation and physical disability by structural equation modeling. The effects of ProMo will be assessed by intention-to-treat principal using repeated measures ANOVA, covariance analysis and linear mixed models for continuous variables and by general estimation equation for categorical variables.

### Characteristics of the participants

Table 1 summarizes the demographics, physical characteristics, health, living habits, fracture status and the type of operation in the total sample and in the intervention and control groups. No significant differences were observed in any variable between the study groups.

**Table 1:** Demographics, health and hip fracture status among over 60-year-old men and women after a recent hip fracture (Mean ± SD, frequency)

	All (n = 76-81)	ProMo (n = 38-40)	Control (n = 38-41)	p*
Age, yr	79 ± 7	80 ± 8	79 ± 6	0.251
Body height, cm	160.6 ± 8.9	160.9 ± 8.9	160.3 ± 9.1	0.785
Body weight, kg	65.8 ± 11.5	65.8 ± 11.9	65.9 ± 11.3	0.968
BMI	25.5 ± 3.8	25.3 ± 3.6	25.6 ± 3.9	0.710
Poor self-rated health, %	41	43	39	0.823
Number of chronic diseases, n	3 ± 2	3 ± 2	3 ± 2	0.581
MMSE	26 ± 3	26 ± 3	26 ± 3	0.686
BDI-II	9 ± 6	9 ± 6	8 ± 6	0.335#
hCRP at baseline	7.7 ± 9.9	8.4 ± 11.1	7.1 ± 8.6	0.855#
Hb at baseline, g/l	128.7 ± 12.9	127.4 ± 12.7	130.1 ± 13.1	0.351
Lowest Hb after operation, g/l	98.0 ± 13.2	97.5 ± 11.1	98.5 ± 15.0	0.795#
Smoking, %				0.382
- Never	79	85	73	
- Former	12	10	15	
- Current	9	5	12	
Living alone, %	59	60	59	1.000
Level of education Elementary school or less, %	49	54	44	0.502
Income, €/month	1363 ± 828	1321 ± 637	1408 ± 998	0.965#
Physical activity, % Sedentary	92	95	90	0.675
Fracture status				
Fall related fracture, %	88	90	85	0.737
Site of fracture, %				0.645
- Femoral neck	64	68	61	
- Pertrochanteric	36	32	39	
Type of operation, n				0.719
- Internal fixation	38	19	19	
- Hemiarthroplasty	33	15	18	
- Total arthroplasty	10	6	4	

*Independent t-test for normally distributed continuous variables, Chi-square for discrete variables

#Mann-Whitney U for non-normally distributed continuous variables

The mean age of the participants was 79 (SD ± 7) years and 78% of the participants were women. More than half of the subjects were living alone. Poor self-rated health was reported by 41% of the total sample. The average number of chronic diseases was 3 ± 2. The mean MMSE value was 26 ± 3 and that of the BDI-II 9 ± 6. Offending pain in lower back, hip or knee region on the fractured side was reported by 46% of the participants. The corresponding value for the non-fractured side was 37%. Nine percent of the participants were current smokers and 92% were rated sedentary.

The majority of the participants (n = 71/81) fell from standing height. Six were able to break the fall e.g. with an outstretched arm. More than half (n = 43) fell indoors and from those 34 participants fell at home. Fifty two participants suffered a femoral neck and 29 a pertrochanteric fracture. Fracture was operated with internal fixation in 38 and with arthroplasty in 43 participant.

## Discussion

The baseline results of our randomized controlled trial emphasize that there is an urgent need to develop long-term rehabilitation strategies for mobility recovery and prevention of mobility disability after hip fracture. According to the patients' own report, only half of them received a home exercise program and followed up the instructions given by the health care personnel on a daily basis. Home exercise programs were not updated and programs did not include any external resistance, walking or balance exercises. Less than 15% of the participants were referred to physiotherapy, while the rest did not get any further instructions or follow-up for recovery of mobility and functional capacity.

Previous studies have shown poor mobility recovery after hip fracture and some of the studies suggest that this phenomenon may turn out to be permanent [1,10,45]. Poor lower limb muscle strength, postural balance and hip pain are associated with poor mobility recovery after hip fracture [10,45]. Muscle strength deficit on the fractured side is associated with greater pain on the fractured compared to the non-fractured side [11] and large muscle strength deficit is associated with mobility limitation and balance impairment [46]. Some recovery is expected to occur during the first six months after hip fracture. However, our earlier study showed that community-dwelling older men and women who had suffered a hip fracture on average four years earlier were significantly weaker, had a significant side-to-side difference in lower limb muscle strength [11,15] and had significantly impaired postural balance and balance confidence [14] compared to the age and sex matched controls with no major lower limb injuries. The presence of multiple impairments, pain and poor balance confidence (fear of falling) strongly suggest increased and cumulative risk for loss of mobility in the near future if targeted rehabilitation with follow-up for mobility recovery is not available.

The standard care, in this study, did not include the follow-up for mobility recovery. It included home exercise programs with five to seven exercises mostly for the fractured limb. Programs were not updated to a more challenging one and no additional resistance was used. None of the participants were followed-up for the home exercise program. Variation in the rehabilitation activities and lack of guidelines for mobility limitation and disability prevention after hip fracture has been recognized worldwide [47,48]. It has been suggested that better functional outcomes could be achieved with more intensive rehabilitation and promotion of physical activity after hip fracture [47,48].

The aim of the ProMo -intervention is to restore mobility after hip fracture and it was firmly grounded to existing research literature. As we wanted to include all hip fracture patients who could potentially benefit from the rehabilitation, also the weakest and the oldest ones, the program was designed to take place at the participants' home. The ProMo is a 1-year progressive physical exercise and physical activity counseling program reinforced by advise, support and encouragement for safe walking as well as discussions on fall prevention and pain management strategies. Pain assessment and fear of falling management was regarded as an essential part of the program as older people who had had a hip fracture suffer from residual pain [12,49] and fear of falling [14]. Both pain [12,50,51] and fear of falling [52-54] have been independently associated with mobility limitation, activity restriction, low physical functioning and falls among older populations. To our knowledge and based on a recently updated Cochrane review [16] there is no previously published effectiveness RCT among community-dwelling hip fracture participants including a home-based intervention specifically targeting on mobility recovery and which has mobility limitation and disability as the main outcome. Encouraging evidence on effects of interventions with similar components on the level of physical activity [22], functional capacity [26] and health related quality of life [55] have, however, been reported.

The recruitment process of this study included eligibility screening in multiple phases and there was close collaboration with clinicians at the local hospital and health care centers. In total, 296 patients who fulfilled the inclusion criteria were identified and informed about the study at the hospital. From those approximately half (n = 161) were interested in and further informed about the study. From those who expressed initial interest 84% (n = 136) signed informed consent and were enrolled in the study. This was regarded as a sufficient number of participants, allowing a 35% attrition rate, ending with 44 in each study group. Because our participants were recruited at the clinic (health care centre) prior to discharge to home, we set our safety margin in the attrition rate higher than 20% which was recommended by Ferrucci et al in their consensus report [56]. We expected some changes in health status, living conditions and willingness to participate to occur already before the baseline measurements. Accordingly, 26% were further excluded due to poor health, alcoholism and living conditions and 15% declined participation mostly due to poor self-rated health and tiredness (Figure 1). Finally, 81 men and women were assessed at baseline and randomly assigned into ProMo -intervention and control groups. Despite of careful planning of the study and target of the recruitment period from 24 to 33 months, we did not completely reach the estimated number needed for this study. However, as the intervention is home-based and individually targeted and the main outcomes can be assessed at the participants' home, we trust that the additional drop-out will be small. The demographics of our study participants is comparable to earlier studies involving community-dwelling older people recovering from hip fracture; the majority of them are women and the mean age is close to 80 [57].

In conclusion, this report summarized the rationale, procedures and intervention of a 1-year RCT with 1-year follow-up on the effectiveness of home-based rehabilitation program aiming to restore mobility after hip fracture among community-dwelling over 60-year-old men and women. The special feature of the current study is that we reinforce the home exercise program by advice, support and encouragement for safe walking and discussions on fall prevention and pain management strategies. In addition, promotion of using existing exercise and rehabilitation services available for older people in their own community was performed by physical activity counseling. These facilities will be available for the participants also after finishing the project. This intervention study will provide knowledge of the rehabilitation for mobility recovery among community-living older people after hip fracture. However, the effectiveness of the program can only be assessed after the end of the study.

## Abbreviations

BDI-II: Beck Depression Inventory; BMI: Body Mass Index; Hb: Hemoglobin; hCRP: C-reactive protein; MMSE: Mini Mental State Examination.

## Competing interests

The authors declare that they have no competing interests.

## Authors' contributions

SS developed the idea of conducting the study, recruited the participants, collected and analyzed the data, interpreted the results and wrote the paper. AS recruited the participants, collected the data and wrote the paper. JE collected the data and wrote the paper. AH conceived the idea of the study, interpreted the results and wrote the paper. MAK analyzed the data and wrote the paper. MA-K conceived the idea of the study, recruited the participants, interpreted the results and wrote the paper. SES collected the data, interpreted the results and wrote the paper. MP conceived the idea of the study, recruited the participants, interpreted the results and wrote the paper. TR conceived the idea of the study, interpreted the results and wrote the paper. MK conceived the idea of the study, recruited the participants, collected data, interpreted the results and wrote the paper. All authors read and approved the final manuscript.

## Pre-publication history

The pre-publication history for this paper can be accessed here:

http://www.biomedcentral.com/1471-2474/12/277/prepub
